# Editorial: Women in behavioral neuroscience: 2022

**DOI:** 10.3389/fnbeh.2024.1374244

**Published:** 2024-02-07

**Authors:** Lisa Y. Maeng, Liana Fattore, Marjorie C. Gondré-Lewis

**Affiliations:** ^1^Department of Psychology, Developmental and Brain Sciences Program, University of Massachusetts Boston, Boston, MA, United States; ^2^CNR Institute of Neuroscience-Cagliari, National Research Council, Cagliari, Italy; ^3^Developmental NeuroPsychoPharmacology Laboratory, Department of Anatomy, Howard University College of Medicine, Washington, DC, United States

**Keywords:** behavioral neuroscience, neurobiology, addiction, stress, anxiety, neurodegenerative disorders, neuropsychiatric disorders

As editors of the Women in Behavioral Neuroscience Research Topic, we are delighted to present this special article collection to highlight the excellence of women from various backgrounds, institutions, and geographical locations, performing top-level research in behavioral neuroscience. Female neuroscientists have long been underrepresented in various aspects of academic life, and a recent report of a gender imbalance in Neuroscience citations raised continuing concerns about fair and accurate evaluation of women's contributions to science as innovators and trailblazers. Contributions from women have been critical to the ongoing growth and discovery within Neuroscience in general, but especially within the dynamic field of Behavioral Neuroscience. Our Research Topic invited female contributors who use behavioral models in their studies of complex neuropsychiatric and neurocognitive phenomena. Behavioral studies provide a window to view and interpret neuronal output in people or animals based on quantifying observations and are invaluable in translating the functional impact of cellular, molecular, or physiological conditions and manipulations. Contributors are leaders in their research areas and mentor the up-and-coming next generation of researchers, both male and female. Their works in this Research Topic involve rodent models of stress and anxiety, compulsivity, social interaction, Alzheimer's disease, addiction, and neuropsychiatric disorders. Authors perform their research in prestigious research institutes and universities located around the world, ranging from the United States (Texas Tech University, Rutgers University, Johns Hopkins University, Louisiana State University, Albert Einstein College of Medicine in New York, University of Rhode Island), Spain (Universitat Jaume I in Castellón de la Plana, University of Almería), and Denmark (University of Copenhagen) to Germany (Philipps-University Marburg) and Belgium (Leuven Brain Institute).

This Research Topic is composed of five original articles that provide novel insight regarding important behavioral domains in different animal models and in both sexes. Rodent models serve an essential role in behavioral/molecular/anatomical work as they facilitate inquiry of important questions at the intersection of brain and behavior, not possible to investigate in humans. Indeed, animal models provide a more rapid route to advances in our understanding of the neural mechanisms and behavioral readouts observed in human neuropsychiatric conditions. However, behavioral tests and outcome measure interpretation need careful consideration to promote internal validity, scientific rigor, and translatability of findings, as we recognize that rodent neuropsychiatric-like behavior, or behavior in general, do not precisely mirror that of humans. Nonetheless, behavioral mechanisms and approaches can provide powerful evidence to extend or refute parallelism for translational impact and will stimulate further investigations.

In this Research Topic, Gaspar et al. explored the source of inconsistencies in studies of aging and anxiety-like behavior. They evaluated whether characteristics of the elevated plus maze (EPM), a commonly used apparatus to measure anxiety-like behaviors, would significantly impact aging effects on study outcomes. They tested 3-month and 24-month-old mice in apparatuses of varying construction characteristics: arm width and material. They reported that certain attributes in the construction of the EPM apparatus, specifically arm width, may significantly affect results, and anxiety-like behavior was more quantifiable within the larger apparatus. They suggested that using the number of open arm entries as a percentage of the total number of entries as a normalization step would provide a more accurate measure of anxiogenic behavior. For aged rats, an increase in open arm entries was more accurately detected with the larger width apparatus consistent with a reduction of anxiety-like behavior with age, whereas no such distinction could be made in the narrow apparatus. These findings suggest features of the EPM apparatus, which are typically overlooked, may potentially influence the ability to detect age-dependent effects in the EPM test.

DiMarco et al. evaluated a commonly used transgenic mouse model of Alzheimer's Disease (AD) that is characterized by amyloidosis, APPswe/PS1dE9, to determine whether unintended acute stress effects from behavioral tests with a stress component such as the Morris Water Maze, induce the cognitive deficits observed in these tasks but not in behavioral tests without stress, such as novel object recognition. Thus, they subjected transgenic and non-transgenic APPswe/PS1dE9 male and female mice to an added stress component such as exposure to predator odor or forced swim stress prior to delayed match-to-position and 3-choice serial-reaction time tasks. They found that neither type of stressor impaired cognitive performance when added to these short-term, non-stress, memory and attention tests, indicating that inherent test stress alone does not explain the inconsistent AD-related cognitive deficits. Together, DiMarco et al. and Gaspar et al. support a closer examination of how aspects of the tools and behavioral tests often used in animal models may impact their outcome measures.

Rodriguez-Borillo et al. investigated if the cerebellum plays a significant generalizable role in activation and regulation of drug-related contextual memory or if its role is limited to cues associated with the single sensory modality, olfaction. This work enhances the notion that the prefrontal-limbic-striatal circuitry is key for storing drug-associated memories and supports a role for the cerebellum within that circuity, given its major direct and indirect projections via the ventral tegmental area (VTA) and its documented activation in other cocaine studies with olfactory cues. The group used a cocaine-induced conditioned place preference procedure paired with tactile cues (textured floors) and assessed neural activation by measuring cFos expression within the posterior cerebellum, the mPFC, NAc, and VTA. They found that cocaine-paired treatment increased expression of cFos in lobules VIII and IX of the posterior cerebellum, as well as within structures of the fronto-striatal-limbic system, independent of locomotor activity. This work should stimulate additional research regarding connectivity and activation of these regions and how the cerebellum is incorporated to function in unison to modulate addiction.

In the study by Bogdan et al., the impact of social environment on genetically driven behavior was investigated. They used an animal model heterozygous (HET) for *Cacnac1c* which encodes the Ca_v_1.2 L-Type calcium channel. In humans, a single nucleotide polymorphism in CACNAC1C confers atypical social behavior and deficits in communication and is linked to autistic behavior. Using a *Cacnac1c* HET rat with deficits in social interaction and communication, the authors measured whether early exposure to wild-type animals via paired housing as juveniles could rescue or attenuate the deficits in social play behavior and in ultrasonic vocalization at 50-kHz. 50-kHz USV is the range emitted during highly rewarding rough-and-tumble play, and indicative of a playful mood. Indeed, HET-WT pairs played more and emitted a greater number of USVs than HET-HET pairs, indicating a potential rescue of the genetic deficit by behavioral social exposure to WT rats. The earlier the pairing of the mixed genotype, the greater the “rescue” of rough-and-tumble play and of the 50-kHz USV emissions. This study has implications that support the role of environment in ameliorating the influence of genetics on social interactions.

Finally, Martín-González et al. used a putative model of compulsivity, namely high-drinker (HD) and low-drinker (LD) animals selected by schedule-induced polydipsia (SIP), to assess the cognitive control and the negative valence system domains in compulsive (HD) rats. The SIP preclinical model proved useful to identify a compulsive vulnerable subpopulation in which to study behavioral and neurochemical alterations of compulsive subjects, thus extending our knowledge of compulsive behavior, a core feature observed in obsessive-compulsive disorder, addiction, bulimia and other neuropsychopathological disorders. The authors characterized the compulsive phenotype selected by SIP by assessing behavioral inflexibility using a probabilistic spatial reversal learning task, motor inhibition and cognitive impulsivity by variable delay-to-signal, and risky decision-making and frustrative non-reward by rodent gambling task. By showing that HD compulsive rats exhibit behavioral inflexibility, insensitivity to positive feedback, waiting impulsivity, risky decision-making, and frustrative non-reward responsiveness, this study highlights the importance of mapping different behavioral domains to prevent, diagnose and treat compulsive spectrum disorders. Ultimately, the findings will likely stimulate research on cognitive and emotional patterns of response in compulsive phenotypes, improve the knowledge of this transdiagnostic trait, and encourage the development of more efficient diagnoses, treatments, and prevention strategies for compulsivity.

We are grateful to the reviewers who meticulously evaluated the scientific merits of the included works. Most of all, we wish to express our gratitude to the authors who shared their research efforts with us. We feel their work will further stimulate investigations in the field, with continued efforts to understand the neurobiology of behavioral addictions, response to stress, emotions and cognition, neuropsychiatric disorders as well as the mechanisms regulating behavioral traits like compulsivity and sociability. In the meantime, we strongly hope the readers will find the collected articles useful and enjoyable, and that our Research Topic could inspire more young women to pursue careers in behavioral neuroscience.

## Author contributions

LM: Writing—original draft, Writing—review & editing. LF: Writing—original draft, Writing—review & editing. MG-L: Writing—original draft, Writing—review & editing.

